# Restoring Balance: Probiotic Modulation of Microbiota, Metabolism, and Inflammation in SSRI-Induced Dysbiosis Using the SHIME^®^ Model

**DOI:** 10.3390/ph18081132

**Published:** 2025-07-29

**Authors:** Marina Toscano de Oliveira, Fellipe Lopes de Oliveira, Mateus Kawata Salgaço, Victoria Mesa, Adilson Sartoratto, Kalil Duailibi, Breno Vilas Boas Raimundo, Williams Santos Ramos, Katia Sivieri

**Affiliations:** 1 Graduate Program in Biotechnology in Regenerative Medicine and Medicinal Chemistry Food, University of Araraquara (UNIARA), Araraquara 14801-340, SP, Brazil; dramarinatoscano@yahoo.com.br (M.T.d.O.); mateus.salgaco@unesp.br (M.K.S.); 2 Graduate Program in Food, Nutrition and Food Engineering, Campus Araraquara, São Paulo State University (UNESP), Araraquara 14800-060, SP, Brazil; fellipe.lopes@unesp.br; 3 Inserm, FPRM, Université Paris Cité, F-75006 Paris, France; victoriamesa@gmail.com; 4 Centro Pluridisciplinar de Pesquisas Químicas, Biológicas e Agrícolas da Universidade Estadual de Campinas (CPQBA-UNICAMP), Paulínia 13148-218, SP, Brazil; adilson@cpqba.unicamp.br; 5 Department of Psychiatry, University of Santo Amaro (UNISA), São Paulo 04743-030, SP, Brazil; kalilduailibi@uol.com.br; 6 Departament of Biological Sciences, Campus Araraquara, São Paulo State University (UNESP), Araraquara 14800-060, SP, Brazil; breno.vilas@unesp.br; 7 Department of Medical Affairs, APSEN Farmacêutica, São Paulo 04753-001, SP, Brazil; williams@bol.com.br

**Keywords:** gut microbiota, SCFAs, anxiety, gut model

## Abstract

**Background/Objectives:** Selective serotonin reuptake inhibitors (SSRIs), widely prescribed for anxiety disorders, may negatively impact the gut microbiota, contributing to dysbiosis. Considering the gut–brain axis’s importance in mental health, probiotics could represent an effective adjunctive strategy. This study evaluated the effects of *Lactobacillus helveticus* R0052 and *Bifidobacterium longum* R0175 on microbiota composition, metabolic activity, and immune markers in fecal samples from patients with anxiety on SSRIs, using the SHIME^®^ (Simulator of the Human Intestinal Microbial Ecosystem) model. **Methods:** The fecal microbiotas of four patients using sertraline or escitalopram were inoculated in SHIME^®^ reactors simulating the ascending colon. After stabilization, a 14-day probiotic intervention was performed. Microbial composition was assessed by 16S rRNA sequencing. Short-chain fatty acids (SCFAs), ammonia, and GABA were measured, along with the prebiotic index (PI). Intestinal barrier integrity was evaluated via transepithelial electrical resistance (TEER), and cytokine levels (IL-6, IL-8, IL-10, TNF-α) were analyzed using a Caco-2/THP-1 co-culture system. The statistical design employed in this study for the analysis of prebiotic index, metabolites, intestinal barrier integrity and cytokines levels was a repeated measures ANOVA, complemented by post hoc Tukey’s tests to assess differences across treatment groups. For the 16S rRNA sequencing data, alpha diversity was assessed using multiple metrics, including the Shannon, Simpson, and Fisher indices to evaluate species diversity, and the Chao1 and ACE indices to estimate species richness. Beta diversity, which measures microbiota similarity across groups, was analyzed using weighted and unweighted UniFrac distances. To assess significant differences in beta diversity between groups, a permutational multivariate analysis of variance (PERMANOVA) was performed using the Adonis test. **Results:** Probiotic supplementation increased *Bifidobacterium* and *Lactobacillus*, and decreased *Klebsiella* and *Bacteroides*. Beta diversity was significantly altered, while alpha diversity remained unchanged. SCFA levels increased after 7 days. Ammonia levels dropped, and PI values rose. TEER values indicated enhanced barrier integrity. IL-8 and TNF-α decreased, while IL-6 increased. GABA levels remained unchanged. **Conclusions:** The probiotic combination of *Lactobacillus helveticus* R0052 and *Bifidobacterium longum* R0175 modulated gut microbiota composition, metabolic activity, and inflammatory responses in samples from individuals with anxiety on SSRIs, supporting its potential as an adjunctive strategy to mitigate antidepressant-associated dysbiosis. However, limitations—including the small pooled-donor sample, the absence of a healthy control group, and a lack of significant GABA modulation—should be considered when interpreting the findings. Although the SHIME^®^ model is considered a gold standard for microbiota studies, further clinical trials are necessary to confirm these promising results.

## 1. Introduction

Anxiety disorders are among the most prevalent psychiatric conditions globally, affecting all age groups, with a lifetime prevalence estimated at 34% in US adults [1], contributing significantly to global morbidity, and being the second leading mental health-related cause of disability-adjusted life years (DALYs) and years lived with disability (YLDs) worldwide [1].

First-line treatments include psychotherapy and pharmacotherapy. Selective serotonin reuptake inhibitors (SSRIs) and serotonin-norepinephrine reuptake inhibitors (SNRIs) are the main first-line recommended pharmacotherapy for anxiety disorders, with small to medium effect sizes compared with a placebo [1], showing some limitations, like their side effects, tolerance, dependence, costs, and individual variation in effectiveness [2].

The gut–brain axis represents a dynamic and bidirectional connection between the gut and the central nervous system. The gut microbiota is a complex ecosystem of microorganisms that can vary significantly between individuals and be influenced by various environmental factors, such as diet, alcohol consumption, and medication use [3].

Regarding medications, SSRIs are among the drugs that have the greatest influence on gut microbiota composition [4]. Many studies have reported microbiota alterations in both humans and mice due to antidepressant intake [5,6,7].

SSRI agents classically act by increasing the amount of serotonin in the synaptic cleft by inhibiting the pumps that direct serotonin back into the presynaptic neuron [8]. In addition to that known mode of action, some studies have demonstrated a notable antimicrobial activity of these drugs, mainly against Gram-positive bacteria such as *Staphylococcus* and *Enterococcus* [9,10,11,12], possibly by blocking the function of efflux pumps and/or disrupting various processes involved in the biosynthesis of products in microorganisms (such as slime production and swarming), interfering with fundamental metabolic activities [9]. Furthermore, SSRIs present activity against potentially anaerobic bacteria, such as *Clostridium perfringens*, *Clostridium difficile*, and toxigenic *Enterobacteria* [9,13].

It seems that intestinal exposure to SSRIs can also enhance the excitability of intrinsic primary afferent neurons in the inter-muscle plexus and alter the richness and evenness of the gut microbiota [14]. Sertraline, a drug widely used in the treatment of anxiety, showed an important decrease in microbial alpha diversity in mice during treatment in this same study [14]. Similarly, escitalopram, another SSRI, can modify the gut composition towards a decreased Bacillota/Bacteroidota ratio and an increase in Prevotellaceae [15,16]. From this perspective, treating anxiety disorders with SSRIs may pose a challenge to gut microbiota. Thus, adjunctive treatments that can mitigate this negative effect on the microbiota may be quite useful. One possibility in this regard would be the use of probiotics in conjunction with antidepressants.

Probiotics are defined as living microorganisms that when consumed in adequate amounts are beneficial for the host [17]. Studies have shown that probiotics can reshape the gut microbiota composition, reducing gut dysbiosis by regulating the abundance of short-chain fatty acids [18], particularly acetate and butyrate, increasing α-diversity, and re-establishing plasma levels of tryptophan and tryptophan metabolites [19,20,21]. Worth mentioning, increased hippocampal levels of neurotransmitters and brain-derived neurotrophic factor (BDNF) are also described as an effect of probiotics, as well as decreased serum corticosterone and adrenocorticotropic hormone and improved neurite outgrowth in the dentate gyrus. [21,22,23,24,25,26,27,28]. One particular probiotic of interest is *Lactobacillus helveticus* R0052 and *Bifidobacterium longum* R0175 in conjunction. Previous studies have demonstrated that this combination can modulate the gut microbiota, in animals and ex vivo models, shifting the gut microbial structure, as revealed by 16S rRNA gene amplicon sequencing [29,30]. Additionally, the increase in gamma-aminobutyric acid (GABA), short-chain fatty acids and anti-inflammatory cytokines (and the reduction in pro-inflammatory ones) were also shown [29].

However, several gaps remain in the current literature regarding the role of probiotics in alleviating selective serotonin reuptake inhibitor (SSRI)-induced gut dysbiosis. Certain *Lactobacillus* and *Bifidobacterium* strains have demonstrated anti-inflammatory properties and the ability to modulate microbiota composition [31]. Still, further research is needed to clarify how specific probiotic strains influence neurotransmitter production, reduce systemic inflammation, and enhance gut barrier integrity in individuals taking SSRIs [32]. Future studies should focus on how probiotics may help restore the symbiotic relationship between the gut microbiota and the brain, which is often disrupted by SSRI use [33]. Although some findings are promising, more extensive research is required to confirm these results and assess the long-term impact on both gut health and mental well-being. A translational gap persists between preclinical studies and clinical applications [34], as many studies report beneficial effects in animal models that do not consistently translate to human populations [35]. Therefore, the aim of the present study is to evaluate the effects of *Lactobacillus helveticus* R0052 and *Bifidobacterium longum* R0175 in patients with anxiety using antidepressants, utilizing the Simulator of the Human Intestinal Microbial Ecosystem model, in order to open future perspectives for add-on treatment, improving the quality of the gut microbiota.

## 2. Results

### 2.1. Microbiota Composition in Long-Term SHIME^®^ Run

**Figure 1:**
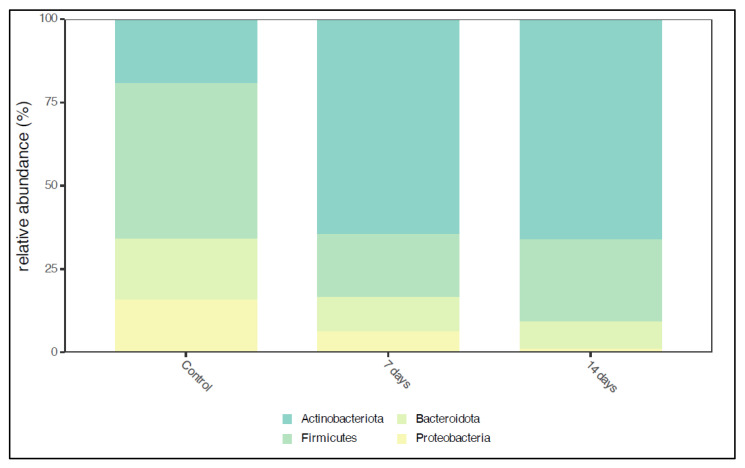
Histogram of gut microbiota composition at the phylum level. Control = control period; 7 days = after 7 days of treatment; 14 days = after 14 days of treatment.

A total of high-quality sequencing reads was obtained from microbiota samples collected during the control period (*n* = 9) and treatment phases (*n* = 9). After normalization, many sequences were retained for further analysis. Figure 1 illustrates the predominant phyla in the microbiota composition before and after 7 and 14 days of treatment. The dominant phyla in the control period were Actinobacteriota, Firmicutes, Bacteroidota, and Proteobacteria. After 7 and 14 days, a significant increase (*p* < 0.01) in Actinobacterioda abundance was observed, while Firmicutes and Proteobacteria showed a slight reduction (Figure 1).

**Figure 2:**
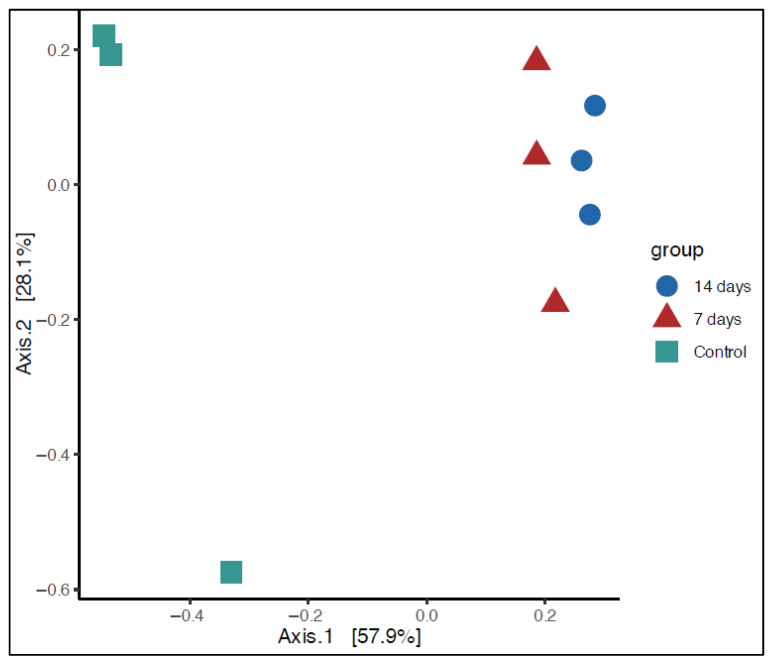
Principal Coordinate Analysis (PCoA) based on microbial community composition using UniFrac distances. Unweighted UniFrac *p* < 0.01. Control = control period; 7 days = after 7 days of treatment; 14 days = after 14 days of treatment.

Alpha diversity analysis showed no significant differences between the control and treatment periods (7 and 14 days). However, beta diversity analysis revealed a statistically significant difference (*p* < 0.01) between the control and treatment groups. Principal Coordinate Analysis (PCoA) demonstrated clear clustering patterns for the control, 7-day, and 14-day groups (Figure 2).

**Figure 3:**
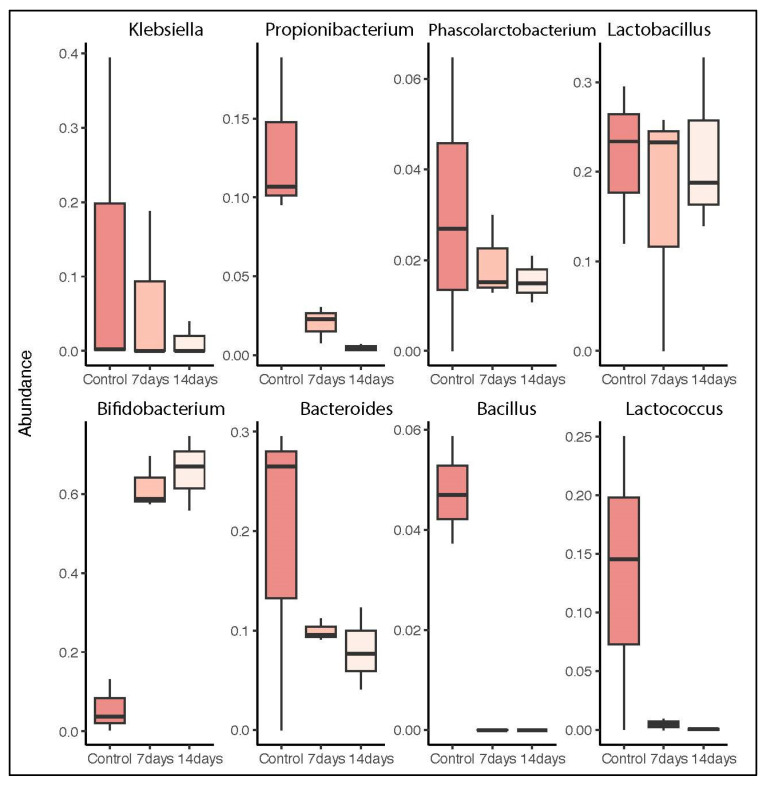
Relative abundance of bacterial genera in the gut microbiota over the course of 7 and 14 days of treatment. Differences in means were analyzed using the Wilcoxon test, *p* < 0.01.

At the genus level, the taxonomic analysis of the microbiota in the SHIME^®^ model showed that after 7 and 14 days of treatment, the relative abundance of *Bifidobacterium* significantly increased (*p* < 0.01). Meanwhile, a significant reduction was observed in the abundance of *Klebsiella* and *Bacteroides* (Figure 3).

**Figure 4:**
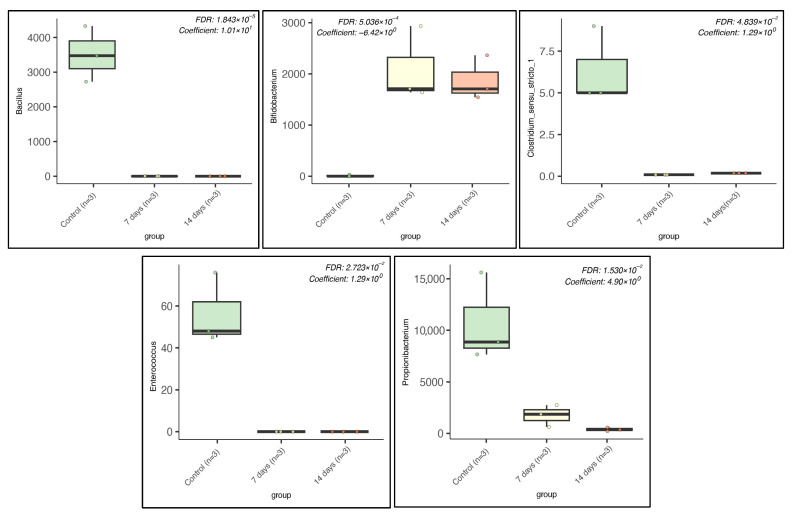
Box plots generated by MaAsLin2 show the differential abundance of microbial species by experimental group. FDR *p*-values and regression coefficients as well as experimental group variables are shown in the upper right corner of each plot.

To further explore the differences in bacteria genera, multivariate analyses using linear models (MaAsLin) were used to find associations between microbiome data and groups. We observed that the relative abundance of some bacteria in the control group was higher than that in the other groups, such as *Bacillus* ssp., *Enterococcus* ssp., *Clostridium* sensu stricto 1, and *Propionobacterium* spp., while the abundance of *Bidobacterium* ssp. was higher after 7 and 14 days of probiotic treatment (Figure 4).

### 2.2. Prebiotic Index (PI)

**Figure 5:**
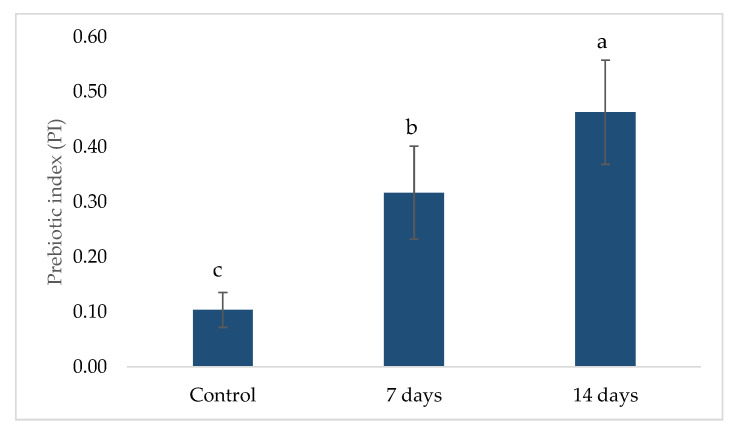
Prebiotic index (PI) variations over control, 7-day, and 14-day probiotic treatment periods. Different lowercase letters represent statistical difference within the phases of the same experiment (*p* < 0.01, ANOVA and post hoc Tukey test). The error bars in the figure represent standard deviation.

The prebiotic index (PI) values obtained during the control, 7-day, and 14-day treatment periods are presented in Figure 5. An increase in the PI was observed following probiotic treatment. During the control phase, the average PI was 0.0315 ± 0.1267, while after 7 days of treatment, it increased to 0.1963 ± 0.2162. The most significant increase occurred after 14 days, reaching 0.3047 ± 0.2816. The PI increase suggests a positive modulation of beneficial bacterial populations, such as *Lactobacillus* (LAC) and *Clostridium* (CLO), alongside a reduction in *Bacteroides* (BAC), which is associated with gut dysbiosis. The trend observed in the 14-day treatment phase indicates a sustained prebiotic effect, supporting the hypothesis that probiotic supplementation enhances beneficial bacterial activity in the gut microbiota.

### 2.3. Metabolic Activity: Ammonia (NH_4_^+^) Production, Short Chain Fat Acids (SCFA) and Gamma-Aminobutyric Acid (GABA)

**Figure 6:**
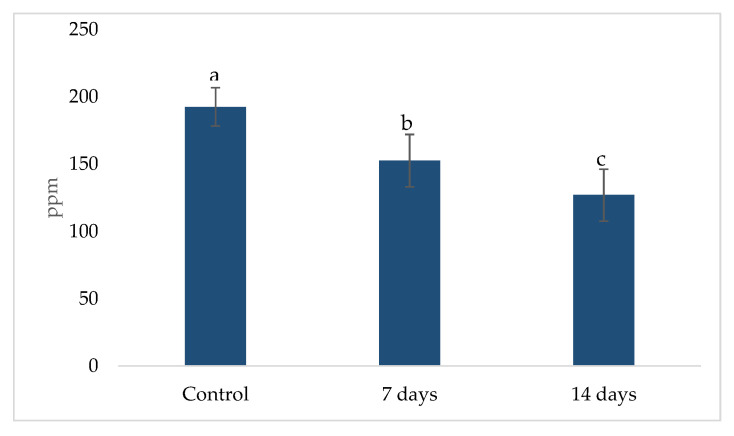
Ammonia (NH_4_^+^) concentration in the ascending colon using the SHIME^®^ model over the course of 7 and 14 days of treatment. Different lowercase letters represent statistical difference within the phases of the same experiment (*p* < 0.01, ANOVA and post hoc Tukey test). The error bars in the figure represent standard deviation.

The metabolic activity related to ammonia (NH_4_^+^) production is shown in Figure 6. A significant reduction (*p* < 0.01) in NH_4_^+^ levels was observed after 7 and 14 days of treatment when compared to the control period. The mean NH_4_^+^ concentration in the control phase was 192.44 ± 14.19, which decreased to 152.44 ± 19.50 after 7 days and further to 126.78 ± 19.32 after 14 days. This reduction suggests a potential modulatory effect of the treatment on ammonia production.

**Figure 7:**
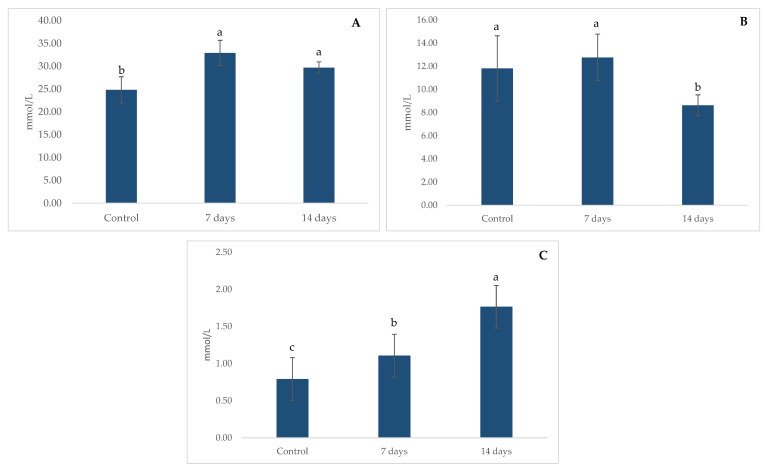
Short-chain fatty acids (SCFAs) concentration in the ascending colon using the SHIME^®^ model over the course of 7 and 14 days of treatment. (**A**) Acetate; (**B**) propionate; and (**C**) butyrate. Different lowercase letters represent statistical difference within the phases of the same experiment (*p* < 0.01, ANOVA and post hoc Tukey test). The error bars in the figures represent standard deviation.

The metabolic activity related to SCFA production is shown in Figure 7. Significant increases (*p* < 0.01) in acetate (A) and butyrate (C) were observed after 7 and 14 days of treatment compared to the control period. However, after 14 days of treatment, a decrease in propionate (B) was observed.

**Figure 8:**
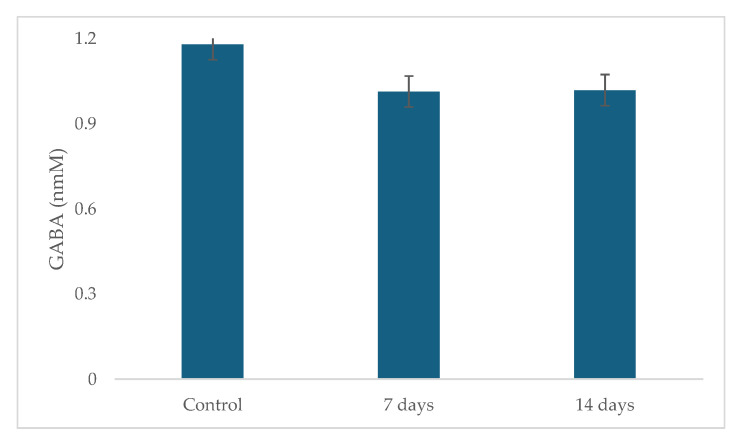
Gamma-aminobutyric (GABA) in the ascending colon using the SHIME^®^ model over the course of 7 and 14 days of probiotic treatment. The error bars in the figure represent standard deviation.

The results of GABA production are shown in Figure 8. During the treatment, GABA production did not increase and was not statistically significant compared to the control period.

### 2.4. Potential Regulation of Gut Epithelial Function and Immune Response

**Figure 9:**
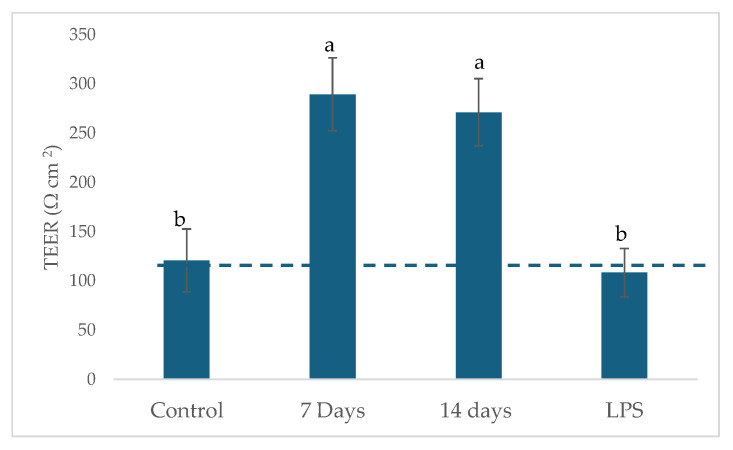
Barrier integrity of Caco-2 cells in the Caco-2/THP coculture system after exposure to SHIME supernatants. Different lowercase letters represent statistical difference within the phases of the same experiment (*p* < 0.01, ANOVA and post hoc Tukey test). The error bars in the figure represent standard deviation. The dashed lines represent the control values.

Samples collected from the ascending colon reactors were used to challenge the Caco-2/THP1 co-culture model. This model enables the assessment of cytokine-mediated disruption of tight junctions. Thus, TEER serves as an indicator of barrier function and monolayer integrity. When compared to the control period, all SHIME^®^ collected samples were able to protect the integrity of the Caco-2 monolayer and showed a significant increase (*p* < 0.01) in TEER (Figure 9).

**Figure 10:**
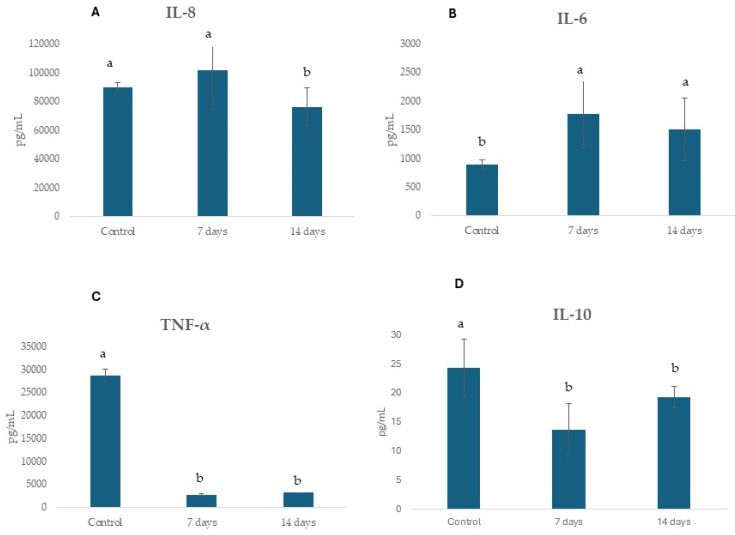
Effect of SHIME supernatants on immune markers in the Caco-2/THP coculture system. (**A**) IL-8, (**B**) IL-6, (**C**) TNF-α, and (**D**) IL-10 activity using SHIME supernatants from 7 and 14 days of probiotic treatment and control period (without treatment). Different lowercase letters represent statistical difference within the phases of the same experiment (*p* < 0.01, ANOVA and post hoc Tukey test). The error bars in the figure represent standard deviation.

Figure 10 shows the concentrations of anti-inflammatory cytokines IL-6, IL-8, and IL-10 and TNF-α following exposure of the cells to colonic media after probiotic treatments (7 and 14 days) for 24 h. The treatment with probiotic decreased IL-10, IL-8, and TNF- α and increased IL-6 secretion.

## 3. Discussion

The present study provides novel insights into the impact of *Lactobacillus helveticus* R0052 and *Bifidobacterium longum* R0175 on the gut microbiota of patients with anxiety undergoing antidepressant treatment, using the SHIME^®^ model. Our findings reveal significant alterations in microbial composition, metabolic activity, and gut epithelial function, suggesting that probiotics may play a crucial role in mitigating SSRI-induced microbiota disruptions and fostering a healthier gut environment.

The taxonomic shifts observed in this study demonstrate a marked increase in Firmicutes and a concurrent reduction in Bacteroidota following probiotic supplementation. At the genus level, the significant rise in *Bifidobacterium*, coupled with the decline in *Klebsiella* and *Bacteroides*, suggests a transition towards a more balanced and resilient gut microbiome. Interestingly, Jiang et al. [36] showed that the microbiota composition of patients with major depressive disorder (MDD) was associated with dysbiosis, characterized by increased *Bacteroidetes* and Proteobacteria and decreased Firmicutes. Therefore, maintaining a dynamic balance of intestinal microbiota within a stable ecological niche is essential for gut homeostasis. Probiotics, such as *Bifidobacterium and Lactobacillus*, contribute to this balance by directly increasing the population of beneficial bacteria while also promoting the growth of endogenous beneficial microbial communities. Additionally, probiotics regulate intestinal homeostasis through competitive exclusion, a natural mechanism in which microorganisms compete for nutrients and ecological niches. This process enhances the colonization of beneficial bacteria while inhibiting the proliferation of pathogenic species.

Despite these compositional changes, alpha diversity remained stable, while the beta diversity analysis revealed distinct clustering patterns between the control and treatment groups. This suggests that while overall microbial richness was maintained, the structural reorganization of the gut microbiome was significantly influenced by probiotic intervention. Antidepressant treatment could modify gut microbiota, which can partially mediate their antidepressant effects. Lukić et al. [7] showed in an animal model that mice chronically treated with one of five antidepressants (fluoxetine, escitalopram, venlafaxine, duloxetine or desipramine) showed a reduced richness of gut bacteria compared to controls.

SCFA production is a key indicator of microbial metabolic activity, with acetate, propionate, and butyrate playing essential roles in maintaining gut homeostasis, modulating inflammation, and influencing brain function via the gut–brain axis [37]. The observed significant increase in acetate and butyrate levels after 7 days and decrease in propionate after 14 days of probiotic supplementation support the hypothesis that probiotics enhance microbial fermentation and metabolic output. However, the subsequent decline at 14 days in propionate suggests a dynamic adaptation process within the gut ecosystem. The transient elevation in short-chain fatty acid (SCFA) levels may reflect an initial microbial shift toward enhanced metabolic activity, followed by regulatory mechanisms that stabilize SCFA production over time. In gut models, such as SHIME^®^, this phenomenon is commonly observed as the gut microbiota gradually adapts to new environmental conditions introduced by probiotics [38]. This adaptation involves complex microbial interactions, including competition for nutrients and cross-feeding mechanisms, where certain bacteria consume SCFAs produced by others, thereby contributing to a reduction in their overall concentration. Similar dynamic patterns have been reported during inulin fermentation, where an initial surge in microbial metabolic response is followed by a progressive stabilization phase [39]. The implications of this trend warrant further investigation, particularly in understanding how sustained probiotic intake influences long-term metabolic equilibrium and its potential effects on gut–brain communication [40].

The integrity of the gut epithelial barrier is crucial for preventing systemic inflammation and maintaining homeostasis. The increased transepithelial electrical resistance (TEER) observed in this study indicates that probiotic supplementation contributed to enhanced barrier function. Several studies cited suggest that *Bifidobacterium* and *Lactobacillus* species have the potential to enhance gut barrier function. For instance, *Bifidobacterium longum* has been shown to protect against intestinal epithelial damage by attenuating inflammatory responses and oxidative stress [41]. More specifically, *Bifidobacterium* strains are known to modulate the expression of tight junction (TJ) proteins, which are critical for maintaining the structural integrity of the intestinal barrier [42]. Dysfunction of TJs is often implicated in the pathogenesis of inflammatory bowel disease (IBD) and other gastrointestinal disorders. By reinforcing this barrier, probiotics may help prevent the translocation of luminal antigens and pathogens into systemic circulation, thereby reducing mucosal and systemic inflammation [43]. In addition, this protective effect may be linked to the upregulation of tight junction proteins, a mechanism frequently associated with butyrate production [44].

Furthermore, the reduction in pro-inflammatory cytokines IL-8 and TNF-α, underscores the immunomodulatory potential of probiotic supplementation. Studies show that probiotics can influence the levels of inflammatory cytokines, thereby modulating the immune response [43]. However, we observed elevated levels of interleukin-6 (IL-6) following probiotic treatment. IL-6 is a pleiotropic cytokine commonly associated with inflammation. Conversely, IL-6 deficiency has been linked to alterations in the composition of the intestinal microbiota [45]. Notably, IL-6 plays a complex and context-dependent role in intestinal inflammation, functioning as both a pro-inflammatory and anti-inflammatory cytokine [46]. In the context of inflammatory bowel disease (IBD), IL-6 has been shown to contribute to chronic intestinal inflammation [47]. However, it can also promote intestinal epithelial cell proliferation and tissue repair following injury [45]. It remains unclear whether IL-6 in our study is acting in a pro- or anti-inflammatory capacity. However, it is possible that the observed increase in IL-6 in response to probiotic treatment may have contributed, at least in part, to the improvement in intestinal membrane permeability.

For instance, *Bifidobacterium longum* has demonstrated anti-inflammatory properties, and its administration can lead to a reduction in pro-inflammatory cytokines [48]. By reducing inflammation, probiotics support overall gut health and can alleviate symptoms associated with inflammatory conditions [42]. Therefore, it is plausible that the probiotics could mitigate some of the negative impacts of SSRIs on the gut microbiota, potentially enhancing intestinal barrier integrity and reducing inflammation, potentially through mechanisms involving SCFA-mediated immune regulation and the modulation of gut microbiota-derived metabolites [49].

While GABA production did not exhibit significant changes following probiotic supplementation in this study, the observed increase in *Bifidobacterium* suggests great potential for gut microbiota modulation. De Oliveira et al. [29] investigated the effects of *Lactobacillus helveticus* R0052 and *Bifidobacterium longum* R0175 on the gut microbiota of mildly anxious adults using the SHIME^®^ model. The participants in that study were not on antidepressants, and an increase in GABA production was found after 14 days of probiotic treatment. The baseline GABA levels during that study’s control period were, however, lower than those in the current study. Given these findings, we speculate that the chronic utilization of antidepressants (longer than one year) in subjects in our study group could have affected basal GABA levels. On the other hand, not all *Bifidobacterium* strains are high GABA producers. While increasing the overall abundance of *Bifidobacterium* [50], the specific strains that flourish might not be those with high glutamic acid decarboxylase (GAD) activity, the enzyme responsible for converting glutamate to GABA [51]. Some *Lactobacillus* and *Bifidobacterium* strains are known to produce neuroactive substances, including GABA, which can influence the gut–brain axis [52]. For example, *Lactobacillus plantarum* strains have been found to produce GABA and exhibit antidepressant [52]. However, an increased abundance of certain *Bifidobacterium* strains may not correspond to high glutamate decarboxylase (GAD) activity, and thus GABA levels may not significantly rise. In addition, GABA production depends on the availability of glutamate, the precursor amino acid [53]. Even if *Bifidobacterium* abundance increases, a limited supply of glutamate could constrain GABA synthesis. The metabolic environment within the gut and the availability of other nutrients also play a crucial role [54].

This in vitro study suggests that *Lactobacillus helveticus* R0052 and *Bifidobacterium longum* R0175 promoted beneficial shifts in the gut microbiota of patients with anxiety undergoing antidepressant treatment, highlighting the potential of probiotic interventions as a complementary therapeutic approach. The gut microbiota plays a central role in the production of key metabolites, including short-chain fatty acids (SCFAs), neurotransmitters, and other neuroactive compounds [55]. SCFAs such as acetate and butyrate can influence brain function by modulating immune responses and exerting direct effects on neuronal signaling. Probiotic supplementation has been shown to alter the metabolic activity of the gut microbiota, leading to changes in SCFA production that may impact anxiety-related symptoms. For example, *Bifidobacterium* strains have been associated with shifts in microbial composition, SCFA metabolism, and immune function [56]. In contrast, gut dysbiosis can promote the overproduction of reactive oxygen species (ROS), contributing to systemic inflammation [57]. This microbiota–brain communication is mediated through several mechanisms, including modulation of neurotransmission, antioxidative responses, and neuroinflammatory pathways [58]. However, the effects of probiotics on anxiety symptoms appear to be strain-specific and are influenced by variables such as dosage, treatment duration, and the host’s baseline microbiota composition [59]. Furthermore, while short-term benefits have been observed, the potential long-term effects of probiotic supplementation on gut microbiota stability and overall health remain underexplored. This is particularly relevant in the context of prolonged SSRI use, which has been associated with persistent alterations in microbial communities. Future studies should investigate whether sustained probiotic intake can maintain or restore microbial homeostasis, reduce chronic inflammation, and support long-term mental health outcomes in individuals undergoing extended antidepressant therapy [60].

This study presents both strengths and limitations that should be considered when interpreting the findings. One of the major strengths is the use of the SHIME^®^ model, a dynamic and physiologically relevant in vitro system that allows for controlled simulation of human colonic fermentation. This model enables a detailed evaluation of the impact of probiotic supplementation on gut microbial composition, metabolic activity, and inflammatory responses under standardized conditions. Furthermore, the integration of multiple analytical approaches, including cytokine profiling, SCFA quantification, and microbial taxonomic shifts, enhances the robustness and comprehensiveness of the results. However, this study has some limitations. The use of a pooled fecal sample from four donors limits the statistical power and generalizability of the results, as it does not account for interindividual variability in microbiota composition and response. Furthermore, the absence of a healthy control group prevents comparative analysis between the microbiota responses of anxious and non-anxious individuals. Although changes in inflammatory markers were observed, no significant increase in GABA levels was detected following probiotic supplementation, suggesting that additional factors or longer exposure durations may be required to modulate neurotransmitter production.

## 4. Materials and Methods

### 4.1. Simulated Digestion in the Dynamic Colonic Model

**Figure 11:**
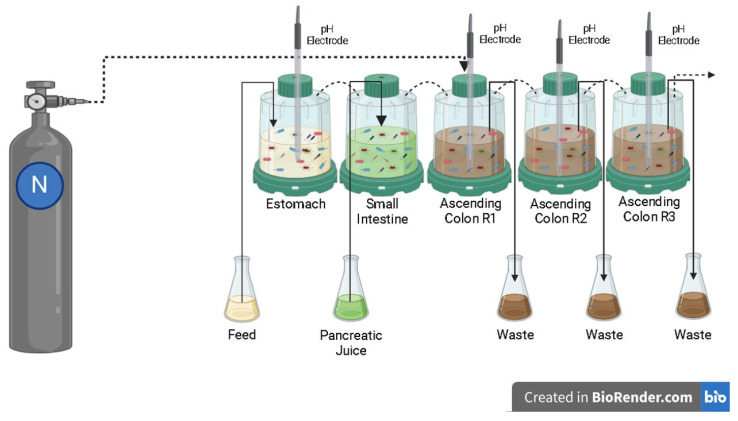
Schematic representation of the Simulator of the Human Intestinal Microbial Ecosystem (SHIME^®^). Created in BioRender. Salgaço, M. (2025) https://BioRender.com/4hm7g3a.

The Simulator of the Human Intestinal Microbial Ecosystem (SHIME^®^) is a dynamic model of the human gastrointestinal tract integrated with software. It consists of five interconnected reactors that represent different sections of the digestive system: the stomach (R1), small intestine (R2), ascending colon (R3), transverse colon (R4), and descending colon (R5). Each reactor maintains specific conditions regarding pH, residence time, temperature, and volumetric capacity. In this study, reactors R4 and R5 were modified to function as R3, enabling a triplicate simulation of the ascending colon, with a pH range of 5.6 to 5.9 and a retention time of 20 h (Figure 11). Stomach conditions were simulated with a pH range of 2.3 to 2.5 and a retention time of 2 h. To mimic duodenal passage, 60 mL of artificial pancreatic juice (containing 12.5 g/L NaHCO_3_, 3.6 g/L Oxgall, 0.9 g/L pancreatin–Sigma-Aldrich, St. Louis, MO, USA) was introduced at a flow rate of 4 mL/min, with a retention time of 4 h [61]. The pH of the reactors was automatically adjusted using sodium hydroxide or hydrochloric acid, and the entire system was maintained at 37 °C. Anaerobic conditions were ensured by injecting nitrogen for 30 min daily [62].

Initially, 500 mL of sterile feed medium was added to the ascending colons [1.0 g/L arabinogalactan (Sigma-Aldrich, St. Louis, MO, USA), 2.0 g/L pectin (Sigma-Aldrich, São Paulo, Brazil), 1.0 g/L xylan (Roth, Karlsruhe, Germany), 3.0 g/L starch (Êxodo, Hortolândia, Brazil), 0.4 g/L glucose (Synth, Diadema, Brazil), 3.0 g/L yeast extract (Neogen, Lansing, MI, USA), 1.0 g/L peptone (Kasvi, Italy), 4.0 g/L mucin (Sigma-Aldrich, St. Louis, MO, USA), and 0.5 g/L cysteine (Sigma-Aldrich, São Paulo, Brazil)) along with 40 mL of pooled fecal inoculum supernatant [59]. Fecal samples were collected and prepared according to the method previously described by Carvalho et al. [63].

The study was conducted in accordance with the guidelines established in the Helsinki Declaration and approved by UNIARA Bioethics Committee (CAAE: 75897323.6.0000.5383). Donor inclusion criteria require individuals aged 20 to 30 years, with high levels of anxiety, to have used selective serotonin reuptake inhibitors (SSRIs) for more than 1 year (Table 1). Exclusion criteria included antibiotic use in the past six months, prebiotic or probiotic consumption, gastrointestinal or metabolic disease medication, and dietary supplement intake in the past three months.

### 4.2. Experimental Protocol

The experiment lasted five weeks using SHIME^®^, divided into: two weeks of microbiota stabilization (300 mL of feed medium once daily) [64], one week of a control period (240 mL of feed medium plus 60 mL of pancreatic juice once daily), and two weeks of probiotic treatment (240 mL of feed medium, 60 mL of pancreatic juice, plus three probiotic pills (*Lactobacillus helveticus* R0052, 3.0 × 10^9^ CFU·log/pill, and *Bifidobacterium longum* R0175, 3.0 × 10^8^ CFU·log/pill, Lallemand Health Solutions Inc., Montreal, QC, Canada)). Samples were collected every seven days after the start of the control period, stored at −20 °C, and the experiment was conducted in biological triplicate.

### 4.3. Metabolic Activity: Ammonia (NH_4_^+^), Short-Chain Fatty Acids (SCFAs) and Gamma-Aminobutyric Acid (GABA) Production

A specific ammonia ion electrode (Model 95-12, Orion) was used for ammonium ion quantification [65]. SCFA analysis (acetic, propionic, and butyric acids) involved centrifuging 2 mL of colonic fermented samples (14,000 rpm for five minutes), storing 1 mL of the supernatant for fatty acid analysis, diluting it 1:1 with MilliQ water, filtering through Millex^®^ (0.45 μm), and injecting into an Agilent gas chromatograph (model HP-6890, Santa Clara, CA, USA) equipped with an Agilent selective mass detector (model HP-5975) and a DB-WAX capillary column (60 m × 0.25 mm × 0.25 μm) under specific temperature conditions. Helium was used as a carrier gas at a flow rate of 1 mL/min. Analytical curves were constructed using stock solutions of acetic, propionic, and butyric acids. Samples were analyzed in triplicate per ascending colon replica before and after treatment. Data were expressed in mmol/g [66].

GABA production was measured using a colorimetric assay with GABA as the standard. Reactions included 40 μL of supernatant, 108 μL of Tris/HCl buffer [160 mM (Sigma-Aldrich, São Paulo, Brazil)], 6.6 μL of 2-mercaptoethanol [100 mM (Sigma-Aldrich, São Paulo, Brazil)], 10 μL of α-ketoglutarate [100 mM (Sigma-Aldrich, St. Louis, MO, USA)], 10 μL of NADP [25 mM (Sigma-Aldrich, St. Louis, MO, USA)], 5.5 μL of ultrapure water, and 20 μL of GABase (Sigma-Aldrich, St. Louis, MO, USA). Absorbance was measured at 340 nm every 15 s for 5 min in 96-well plates, and the results were expressed in mM of GABA [67].

### 4.4. Microbiological Analysis Employing 16S rRNA Gene Sequencing

Bacterial DNA extraction was performed using the DNeasy^®^ PowerSoil^®^ Pro Kit (QIAGEN, Hilden, Germany) per the manufacturer’s instructions. DNA samples were frozen at −20 °C until molecular analysis. The library was prepared using primers for the V3–V4 region of 16S rRNA (~470 bp, amplified with primers 341F × 806R), and bacterial amplicons were sequenced on the Illumina platform (Novaseq6000 PE 250, Illumina, Inc., San Diego, CA, USA). Sequence processing and analysis were conducted using QIIME2 (https://qiime2.org, accessed on 1 December 2024) [68].

Sequence data were processed and analyzed using QIIME (Quantitative Insights Into Microbial Ecology, version 2022.2.0, https://qiime2.org, accessed on 1 December 2024). On average, each sample yielded 181,443 raw reads. During the demultiplexing and trimming steps, low-quality reads (e.g., those below Q30), sequences of inadequate length, and chimeric reads were filtered out using QIIME [69]. Following these processing steps, the dataset retained an average of 27,154 high-quality reads per sample. These clean reads were then used to define Amplicon Sequence Variants (ASVs).

To identify the taxa present in the samples, a predictive model for the V3 and V4 regions was applied, utilizing the SILVA 138 database (99% OTUs from the 515F/806R sequence region). Operational taxonomic units (OTUs) were clustered based on 99% sequence similarity, and taxonomy was assigned using the SILVA 138 reference database (https://www.arb-silva.de/, accessed on 1 December 2024). Heatmaps and bar plots displaying the relative abundance of OTUs were generated using Python (version 3.7) with scripts developed by ByMyCell Inova Simples Ltda. (Ribeirão Preto, Brazil).

Rarefaction curves were constructed using QIIME. Alpha diversity was assessed using various metrics, including the Shannon, Simpson, and Fisher indices to represent species diversity, while the Chao1 and ACE indices were used to estimate species richness. Beta diversity, which evaluates microbiota similarity across different groups, was analyzed using weighted and unweighted UniFrac distances. To determine significant differences in beta diversity between groups, permutational multivariate analysis of variance (PERMANOVA) was performed using the Adonis test.

Predictive functional profiling of bacterial communities was conducted using Phylogenetic Investigation of Communities by Reconstruction of Unobserved States 2 (PICRUSt2, version 2.4.2). OTUs exported from QIIME2 in standard format were imported into PICRUSt2 for further analysis. Genomic data exploration was carried out in Python (version 3.7) [70].

### 4.5. Prebiotic Index (PI)

Total bacterial load and selected members of gut microbiota are enumerated at baseline (end of the control period) and at the end of the colonic fermentation by plate count, as previously described [71]. To quantify the prebiotic effect, samples are taken in duplicate from the colonic batch vessels with and without treatment at different time points. The Prebiotic Index (PI) is calculated as follows:

$$Prebiotic\;Index\;(PI)=\left(\frac{Bif}{Total}\right)-\left(\frac{Bac}{Total}\right)+\left(\frac{Lac}{Total}\right)-\left(\frac{Clos}{Total}\right)$$

Specifically, Bif is *Bifidobacterium* spp. numbers after the fermentation minus numbers at baseline, Bac is *Bacteroides* spp. numbers after the fermentation minus numbers at baseline, Lac is *Lactobacillus* numbers after the fermentation minus numbers at baseline, Clos is *Clostridium perfringens* numbers after the fermentation minus numbers at baseline, and Total is total bacteria numbers after the fermentation minus numbers at baseline. The prebiotic index equation assumes that an increase in the populations of bifidobacteria and/or lactobacilli is positive, while an increase in bacteroides and clostridia (histolyticum subgroup) is negative. The PI equation offers the advantage of normalizing the bacterial population changes in relation to the initial microbial levels, accounting for the physiological variability that characterizes the experimental process of the in vitro fermentation.

### 4.6. Co-Culture of Caco-2 and THP 1 Cells

Cell Culture: The Caco-2 cell line (Human epithelial cells BCRJ: 0059) and THP-1 cell line (Human mononuclear cells BCRJ: 0234) were maintained in DMEM medium (Gibco, São Paulo, Brazil) with 20% fetal bovine serum (FBS) and RPMI 1640 medium (Gibco, São Paulo, Brazil) with 10% FBS, respectively, both supplemented with 0.1% gentamicin. The culture was carried out in specific cell culture flasks, maintained in an incubator at 37 °C and 5% CO_2_. Culture medium changes were performed every other day [72].

Cell Viability Assay: Approximately 3×10^5^ Caco-2 cells were seeded in 12-well plates and incubated for 24 h at 37 °C in a 5% CO_2_ atmosphere. After this period, the cells were treated with microbiota-derived supernatants at a 1:20 dilution and maintained in culture for 72 h. After incubation, the cells were collected and stained with Fixable Viability Dye eFluor™ 450 (FVS450) at a 1:2000 dilution in PBS (Phosphate-Buffered Saline–Gibco, São Paulo, Brazil). The percentage of FVS450-negative cells was assessed by flow cytometry (Cytoflex, Beckman Coulter, Rio de Janeiro, Brazil). Cell viability was determined using the CytExpert 2.6 software.

THP-1 Cell Differentiation Process: For the differentiation process, THP-1 cells were treated with 50 ng/mL PMA (Phorbol 12-myristate 13-acetate) for three days. Subsequently, the cells were incubated in fresh medium without PMA for 24 h. After this period, co-culture assays with Caco-2 and THP-1 cells were conducted.

Co-Culture System: Human epithelial Caco-2 cells were cultured at a density of 1.5 × 10^5^ cells per well in Transwell inserts (0.4 μm pore size; Greiner Bio-One, Frickenhausen, Germany) and maintained for 21 days at 37 °C in a 5% CO_2_ atmosphere. The culture medium was renewed every three days until the cells reached complete differentiation, verified by measuring transepithelial electrical resistance (TEER) values above 300 Ω⋅cm^2^. Simultaneously, THP-1 cells were seeded in 24-well plates at a concentration of 5 × 10^5^ cells per well in the lower (basolateral) chamber. Subsequently, the insert containing the differentiated Caco-2 monolayer was transferred to the Transwell plate already containing THP-1 cells. To investigate the anti-inflammatory potential of the tested compound, the microbiota supernatant was diluted at a 1:20 ratio in 500 μL of DMEM medium and added to the apical compartment, maintaining the co-culture for 24 h. Then, Caco-2 cells were exposed to 100 ng/mL LPS for another 24 h. At the end of the incubation, the culture medium from the basolateral chamber containing THP-1 cells was collected for the analysis of inflammatory mediator production [73,74].

TEER: Measurements were performed to evaluate the integrity of the cell monolayer using a voltmeter (Millicell-ERS, Millipore, MA, USA). The electrical resistance of each well was measured in triplicate, and the average value obtained was used for all samples. As a control, an insert without cells was used as a blank reference. The TEER value was calculated using the following equation: TEER (Ω⋅cm^2^) = (Resistance − Blank resistance (Ω)) × Membrane surface area (cm^2^) [75].

Cytokine Quantification: After the completion of the co-culture experiment, the supernatant was collected for cytokine quantification produced by differentiated THP-1 cells. The quantification was performed using the ELISA method with IL-6 kits (BD Biosciences (San Jose, CA, USA), detection limit 300: 4.7 pg/mL), TNF-α (BD Biosciences, detection limit 500: 7.8 pg/mL), IL-10 (BD Biosciences, detection limit 500: 7.8 pg/mL), and IL-8 (BD Biosciences, detection limit 200: 3.1 pg/mL). Absorbance at 450 nm was measured using an Epoch plate reader (Biotek, Winooski, VT, USA) [65].

### 4.7. Statistical Analysis

Data were expressed as mean ± standard deviation (SD). Statistical significance was assessed using paired *t*-tests, one-way analysis of variance (ANOVA), and Tukey’s test, with a significance threshold of *p* < 0.05. Analyses were conducted using GraphPad Prism software, version 8.0 (La Jolla, CA, USA).

The 16S rRNA gene sequence analyses were performed in RStudio, version 3.2.4 (R Core Team, https://www.R-project.org, accessed on 1 December 2024), utilizing the phyloseq package [63] to import sample data and compute alpha and beta diversity metrics. The statistical significance of categorical variables was determined using the non-parametric Wilcoxon test for two-group comparisons and the Kruskal–Wallis test for comparisons involving three or more groups. Principal coordinate analysis (PCoA) plots were generated based on the PERMANOVA test to estimate *p*-values. Multiple comparisons were adjusted using the false discovery rate (FDR) algorithm [65,76]. Multivariable association discovery was performed using MaAsLin 2 analysis [77]. The MaAsLin 2 analysis was performed with the fixed_effect=c (“group”).

## 5. Conclusions

This in vitro study provides preliminary evidence that the probiotic combination of *Bifidobacterium longum* R0175 and *Lactobacillus helveticus* R0052 may modulate gut microbiota composition, metabolic activity, and intestinal barrier function in fecal samples from individuals with anxiety undergoing SSRI treatment. The intervention was associated with favorable microbial shifts, including an increase in *Bifidobacterium* a genus often linked to gut health and a reduction in potentially pro-inflammatory genera such as Klebsiella and Bacteroides, which are commonly associated with dysbiosis. These alterations suggest a potential movement toward a more balanced microbial ecosystem, although the limited sample size precludes definitive conclusions.

Improvements in gut barrier function, as indicated by increased transepithelial electrical resistance (TEER), and enhanced production of short-chain fatty acids (SCFAs), particularly acetate and butyrate, further support the potential of this probiotic formulation to contribute to intestinal homeostasis. The reduction in ammonia levels also points to a shift toward a healthier metabolic profile.

While changes in cytokine levels, including reductions in IL-8 and TNF-α, and an increase in IL-6 were observed, these responses were variable and should be interpreted with caution. The complexity of host–microbiota–immune interactions, particularly in the context of SSRI-induced dysbiosis, cannot be fully captured in in vitro systems.

Taken together, these findings suggest that this probiotic combination may hold promise as a complementary strategy to mitigate gut dysbiosis associated with antidepressant use. However, further research including well-powered clinical trials and advanced mechanistic studies is essential to confirm these observations and to better understand the long-term implications for gut and mental health.

### Commentary of Expert (Psychiatrist, Master in Neuropsychiatry and Behavioral Sciences and PhD in Tropical Medicine)

In everyday clinical practice, pharmacological combinations are frequently employed in the treatment of various mental disorders. The complexity of the etiopathogenesis and pathophysiology of these conditions necessitates the integration of psychotherapeutic, sociotherapeutic, and pharmacological approaches, as well as biological interventions such as electroconvulsive therapy or transcranial magnetic stimulation.

In the management of anxiety disorders, clinicians typically rely on a combination of psychotherapy and pharmacotherapy. Among pharmacological options, selective serotonin reuptake inhibitors (SSRIs) and serotonin-norepinephrine reuptake inhibitors (SNRIs) have demonstrated significant therapeutic value.

Recent findings suggest that intestinal dysbiosis may contribute to the etiopathogenesis of anxiety, thereby opening a promising avenue for therapeutic intervention. In particular, strains such as *Lactobacillus helveticus* R0052 and *Bifidobacterium longum* R0175 have shown clinical potential, often used as adjuncts to conventional antidepressants.

Moreover, the observation that serotonergic antidepressants—especially SSRIs—can alter gut microbiota composition reinforces the rationale for co-administering probiotics to help restore microbial balance. This article, using the SHIME^®^ in vitro model, demonstrates improvements in intestinal dysbiosis among patients with anxiety disorders treated with SSRIs. These findings suggest that adjunctive probiotic therapy may offer clinical benefits by enhancing inflammatory regulation, metabolic function, and microbiota composition. The observed synergistic effects of SSRIs (such as sertraline or escitalopram) and probiotics highlight a translational bridge between in vitro findings and in vivo clinical applications. These results support the need for further studies exploring the co-administration of antidepressants and psychobiotics to optimize mental health outcomes.

## Figures and Tables

**Table 1:** Participant Characteristics.

Evaluated Parameters	4 Participants
Hamilton Scale	Severe anxiety
Medications	Escitalopram and Setraline
Sex	2 women and 2 men
Age	36.00 ± 5.41 years old
Weight	64.20 ± 13.20 kg

## Data Availability

Data presented in this study is contained within the article. Further inquiries can be directed to the corresponding author.
